# Rhoa/ROCK, mTOR and Secretome-Based Treatments for Ischemic Stroke: New Perspectives

**DOI:** 10.3390/cimb46040219

**Published:** 2024-04-18

**Authors:** Elena Anca Pinoșanu, Denisa Pîrșcoveanu, Carmen Valeria Albu, Emilia Burada, Andrei Pîrvu, Roxana Surugiu, Raluca Elena Sandu, Alina Florina Serb

**Affiliations:** 1 Department of Neurology, University of Medicine and Pharmacy of Craiova, St. Petru Rares, No. 2-4, 200433 Craiova, Romania; elenapinosanu@yahoo.com (E.A.P.); denisa.pirscoveanu@umfcv.ro (D.P.); carmen.albu@umfcv.ro (C.V.A.); 2 Doctoral School, University of Medicine and Pharmacy of Craiova, St. Petru Rares, No. 2-4, 200433 Craiova, Romania; 3 Department of Physiology, University of Medicine and Pharmacy of Craiova, St. Petru Rares, No. 2-4, 200433 Craiova, Romania; emilia.burada@umfcv.ro; 4 Dolj County Regional Centre of Medical Genetics, Clinical Emergency County Hospital Craiova, St. Tabaci, No. 1, 200642 Craiova, Romania; andrei.crgm@gmail.com; 5 Department of Biochemistry, University of Medicine and Pharmacy of Craiova, St. Petru Rares, No. 2-4, 200433 Craiova, Romania; roxana.surugiu@umfcv.ro; 6 Department of Biochemistry and Pharmacology, Biochemistry Discipline, “Victor Babes” University of Medicine and Pharmacy, Eftimie Murgu Sq., No. 2, 300041 Timisoara, Romania; aserb@umft.ro

**Keywords:** ischemic stroke, RhoA/ROCK, secretome, EVs, mTOR, therapy, cerebral ischemia

## Abstract

Ischemic stroke triggers a complex cascade of cellular and molecular events leading to neuronal damage and tissue injury. This review explores the potential therapeutic avenues targeting cellular signaling pathways implicated in stroke pathophysiology. Specifically, it focuses on the articles that highlight the roles of RhoA/ROCK and mTOR signaling pathways in ischemic brain injury and their therapeutic implications. The RhoA/ROCK pathway modulates various cellular processes, including cytoskeletal dynamics and inflammation, while mTOR signaling regulates cell growth, proliferation, and autophagy. Preclinical studies have demonstrated the neuroprotective effects of targeting these pathways in stroke models, offering insights into potential treatment strategies. However, challenges such as off-target effects and the need for tissue-specific targeting remain. Furthermore, emerging evidence suggests the therapeutic potential of MSC secretome in stroke treatment, highlighting the importance of exploring alternative approaches. Future research directions include elucidating the precise mechanisms of action, optimizing treatment protocols, and translating preclinical findings into clinical practice for improved stroke outcomes.

## 1. Introduction

According to the Global Burden of Disease (GBD 2019), stroke is the second leading cause of death and the third most significant cause of both death and disability, with a significant increase in disability-adjusted life years (DALYs) caused by risk factors, particularly in low- and middle-income countries [1,2].

Strokes are classified into ischemic, hemorrhagic, and transient ischemic attacks (TIAs), with ischemic strokes constituting the majority at approximately 87%. Unlike hemorrhagic strokes, which are characterized by bleeding in the brain, ischemic strokes occur due to blocked blood flow to the brain. TIAs, or “mini-strokes”, present brief, reversible symptoms, acting as precursors to more severe strokes. This review included the articles that focuses on ischemic strokes [3]. The effectiveness of revascularization treatments, including both intravenous thrombolysis and endovascular interventions, is closely linked to time, highlighting the importance of research on other treatment options [4]. Typically, ischemic stroke is identified through exclusion in the emergency setting, with non-contrast computed tomography (NCCT) serving as the primary diagnostic tool. This preference for NCCT is due to its broad accessibility and the relatively quick duration required for imaging, making it a critical initial step in assessing patients suspected of ischemic stroke [5].

In addition to treatment considerations, it is imperative to understand the underlying pathophysiological mechanisms driving ischemic stroke. Ischemic strokes arise from the sudden interruption of blood flow to the brain, precipitating a cascade of neurological deficits, and stands out as the predominant subtype among all stroke occurrences, constituting over 80% of total cases [6]. This interruption leads to cellular damage due to oxygen and nutrient deprivation, triggering inflammatory responses, oxidative stress, and ultimately neuronal death [7]. Key contributors to ischemic stroke pathology include the disruption of blood–brain barrier integrity, excitotoxicity, mitochondrial dysfunction, and apoptotic pathways [8,9].

These pathophysiological mechanisms not only affect individual neurons but also disrupt the intricate balance within the neurovascular unit (NVU). The NVU comprises neurons, astrocytes, endothelial cells, pericytes, and the extracellular matrix, working in concert to maintain brain homeostasis and regulate cerebral blood flow [10].

Despite extensive progress in understanding the ischemic cascade, an effective neuroprotective therapy that safeguards neurovascular units (NVUs) and significantly enhances neurological functions in ischemic stroke patients remains elusive [10,11]. While intravenous thrombolysis and other recanalization therapies show potential in restoring perfusion and improving clinical outcomes if administered promptly after ischemic stroke onset [12], the ultimate aim of preserving NVU integrity and optimizing neurological recovery remains a pressing challenge in stroke care [13].

While numerous cellular signaling pathways contribute to ischemic stroke pathology, this review concentrates on the RhoA kinase pathway and mTOR (mammalian target of rapamycin) as promising therapeutic targets, as well as secretome-based treatments, which have the potential to modulate multiple aspects of stroke pathology and potentially complement existing therapeutic approaches.

The Rho kinase pathway has been associated with the pathophysiology of diverse central nervous system (CNS) disorders and has garnered attention as a promising therapeutic target for ischemic stroke treatment [14]. ROCK (Rho-associated protein kinase), a principal downstream mediator of RhoA activation, participates in a spectrum of intracellular signaling cascades, encompassing the modulation of endothelial nitric oxide synthase (eNOS) expression, blood–brain barrier (BBB) integrity, neuronal apoptosis, and astrogliosis [15]. Moreover, mTOR, a highly conserved protein kinase, plays a central role in various signaling pathways critical for fundamental cellular processes such as cell growth and metabolism [16]. A thorough understanding of the molecular mechanisms activated in response to cerebral ischemia holds significant potential for the identification of novel therapeutic targets. In this context, mTOR emerges as a compelling candidate for inclusion in intervention strategies aimed at ameliorating ischemic stroke pathology [17].

Additionally, we explore the emerging concept of the “secretome”, comprising the bioactive molecules secreted by mesenchymal stromal cells (MSCs), and its potential implications for ischemic stroke treatment. Through an in-depth analysis of these signaling pathways and their modulation, we aim to provide insights into novel therapeutic strategies aimed at attenuating neuronal injury, promoting tissue repair, and enhancing functional recovery following ischemic stroke.

## 2. Search Strategy

A comprehensive literature search was performed across various electronic databases, such as PubMed, Web of Science, Scopus, and Google Scholar, to identify studies relevant for our topic. The search strategy employed a combination of keywords and phrases related to ischemic stroke, molecular mechanisms, pharmacological treatments, RhoA/ROCK, mTOR pathways, MSCs, EVs, and secretome. Boolean operators (AND, OR) were applied to refine the search results. For example, search terms included “stroke OR cerebral ischemia AND RhoA/ROCK”, “MSC OR EVs OR secretome AND stroke”, and “mTOR AND stroke OR cerebral ischemia AND treatment”.

## 3. The Neurovascular Unit and Ischemic Cascade

The ischemic cascade denotes a sequence of intricate biochemical and molecular processes that occur in the brain after an abrupt cessation of blood circulation, commonly caused by an ischemic stroke or other cerebrovascular incidents. This sequence of events takes place as a result of the lack of oxygen and glucose, which are vital for the brain’s energy metabolism and cellular activities [18,19]. Energy depletion causes the malfunction of ion pumps, specifically the sodium–potassium ATPase pump, leading to the depolarization of neurons [20]. The depolarization initiates an excessive release of excitatory neurotransmitters, such as glutamate, resulting in the overstimulation of glutamate receptors, specifically N-methyl-D-aspartate (NMDA) receptors [21,22].

Excitotoxicity and energy failure result in the entry of calcium ions into neurons through several channels, such as NMDA receptors [23] and voltage-gated calcium channels [24]. The excessive buildup of calcium within cells triggers the activation of several enzymes and pathways that lead to cellular destruction, such as proteases, lipases, and nitric oxide synthase [25]. This results in the generation of reactive oxygen species (ROS) and free radicals [26,27], highly reactive compounds that cause oxidative harm to lipids, proteins, and DNA in brain cells, increasing neuronal damage [28,29].

**Figure 1:**
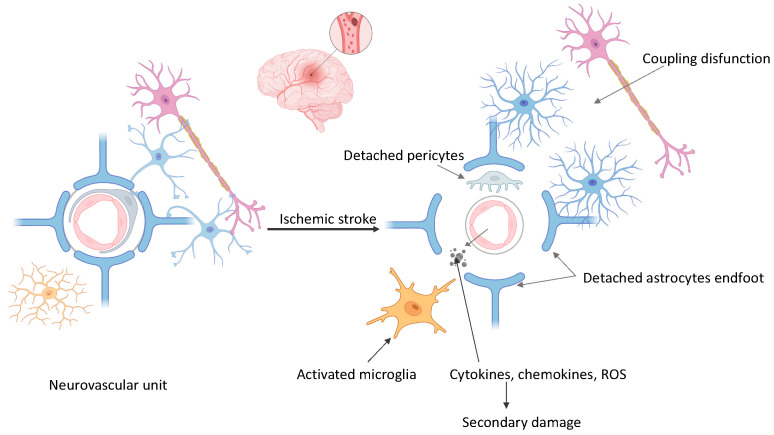
Neurovascular unit changes secondary to ischemia (created with BioRender.com (accessed on 10 March 2024)).

Ischemic brain injury triggers a series of processes characterized by the activation of microglia [30,31,32], astrocytes [33,34,35], and immune cells that infiltrate the brain [36]. The inflammatory reaction triggers the release of cytokines, chemokines, and other substances that promote neuroinflammation and cause more tissue harm [37]. Simultaneously, oxidative stress and inflammation [7] caused by ischemia weaken the blood–brain barrier (BBB), increasing its permeability and enabling the infiltration of chemicals from the bloodstream into the brain tissue [8]. This breach of BBB integrity promotes neuronal damage and inflammation, and together with the combination of excitotoxicity, oxidative stress, and inflammation leads to secondary damage to neurons and tissues, which spreads beyond the original area of reduced blood flow into the surrounding region known as the penumbra [9,38]. This additional insult exacerbates the advancement of ischemia damage, intensifying neurological impairments and compromising results [39] (Figure 1).

## 4. Targeting Cellular Pathways in Ischemic Stroke Treatment

### 4.1. RhoA/ROCK Pathway

RhoA is a small GTPase protein that serves as a molecular switch in intracellular signaling [40], crucial for controlling a range of cellular activities, such as cytoskeletal dynamics, cell adhesion, movement, and growth [41]. RhoA transitions from an inactive GDP-bound form to an active GTP-bound form [42], with its activation being carefully controlled by guanine nucleotide exchange factors (GEFs) [43,44] and GTPase-activating proteins (GAPs) [45]. When triggered, RhoA influences its impact by engaging with downstream effector proteins like ROCK (Rho-associated protein kinase). ROCK functions as a key downstream effector of RhoA, operating as a serine/threonine kinase [46]. It plays a crucial role in various cellular reactions triggered by RhoA activation, such as rearranging the actin cytoskeleton, causing cell contraction, and promoting cell movement [47]. Controlling these processes involves the phosphorylation of different target proteins by ROCK, including myosin light chain (MLC) and LIM kinase (LIMK), which then impact actomyosin contractility and cytoskeletal dynamics [48].

The RhoA/ROCK pathway is an essential signaling path involved in multiple biological processes such as cell proliferation, migration, and contraction [49,50,51]. The RhoA/ROCK pathway is important in regulating various pathophysiological processes that lead to ischemic brain damage in stroke [52].

**Figure 2:**
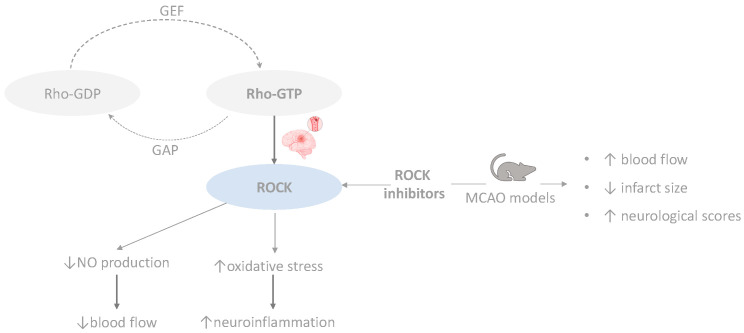
Overview of Rhoa/ROCK pathway in ischemic stroke (created with BioRender.com (accessed on 10 March 2024)). GEF, guanine nucleotide exchange factors; GAP, GTPase-activating proteins.

The impact of the pathway on cerebral ischemic injury is multifaceted and intricate [53]. Activation of the RhoA/ROCK pathway in the vascular endothelium following ischemic stroke may lead to decreased phosphorylation of nitric oxide synthase 3 (NOS3), resulting in reduced nitric oxide (NO) production, which leads to a decreased blood flow [54]. Also, activation of the RhoA/ROCK pathway precipitates a cascade of events characterized by heightened oxidative stress and elicitation of inflammatory responses, thereby accelerating the process of neuroinflammation [55]. Studies using middle cerebral artery occlusion (MCAO) models have shown that inhibiting ROCK results in increased blood flow, leading to a significant reduction in cerebral infarct size and an improvement in neurologic deficit scores [56], as well as enhanced learning capacity [57]. Nonselective pharmacological inhibitors and the lack of understanding regarding downstream targets have limited research on the RhoA/ROCK pathway in ischemic stroke. While ROCK inhibitors like Fasudil have shown promise in preclinical and clinical trials [58], concerns remain about their adverse effects and the need for tissue-specific targeting to dissect the precise role of ROCK isoforms in ischemic stroke pathophysiology [53]. Figure 2 provides an overview of the impacts of the RhoA/ROCK pathway in ischemic stroke and outlines its potential therapeutic implications.

### 4.2. mTOR Signaling Pathway

The mammalian target of rapamycin (mTOR) pathway is a key signaling pathway involved in the regulation of various cellular processes, including cell growth [59], proliferation [60], survival [61], metabolism [62], and autophagy [63,64]. mTOR stands as a highly conserved serine/threonine protein kinase found within the PI3K (phosphoinositide 3-kinase)-related kinase family (PIKK) [65], a class of intracellular lipid kinases that catalyze the phosphorylation of the 3′-hydroxyl group on the inositol ring of phosphatidylinositides [60].

It has been demonstrated that cerebral ischemia causes a significant activation of signaling pathways that are associated with autophagy in neurons, glial cells, and brain microvascular cells [66]. In this context, the classic type I PI3K/Akt–mTOR and AMPK–mTOR pathways play pivotal roles in regulating autophagy after cerebral ischemia [23]. Phosphoinositide 3-kinase (PI3K) activation leads to the phosphorylation and activation of Akt, which subsequently activates TORC1, which inhibits autophagy activation in nutrient-rich environments [67], through Unc-51-like autophagy activating kinase 1 (Ulk-1) [68]. Under ischemic conditions, the activity of this pathway is diminished [69]. Secondly, the AMPK (5′-AMP-activated protein kinase)–mTOR pathway acts as a metabolic sensor, modulating cellular energy balance and autophagic activity in response to energy stress [70]. Ischemic stroke triggers energy depletion and metabolic stress [71], leading to AMPK activation [72]. Activated AMPK inhibits mTORC1 activity, relieving its suppression of autophagy [73]. Enhanced autophagy, facilitated by AMPK activation, promotes the clearance of damaged cellular components and may contribute to neuronal survival and recovery in ischemic stroke [74,75], attenuates neuroinflammation by suppressing the activation of inflammasomes and modulating the phenotypic alteration of microglia [76], as well as promotes blood-brain barrier (BBB) preservation [77].

Nevertheless, the evidence regarding the dual role of autophagy following ischemic stroke is inconclusive. Some studies suggest that heightened autophagic activity exacerbates brain injury in ischemic conditions [78], leading to increased oxidative stress [79] and apoptosis [80].

Several pharmacological agents that activate AMPK have been studied extensively. Pretreatment with metformin has the capacity to regulate mitochondrial biogenesis and pathways associated with apoptotic cell death through the activation of AMPK in a rat model of global cerebral ischemia, thereby conferring neuroprotection [81]. Furthermore, Zhao et al. [82] investigated the impact of metformin on neurological function and oxidative stress in individuals with type 2 diabetes mellitus experiencing acute stroke and found that patients treated with metformin exhibited a significant decrease in NIHSS scores compared to those receiving insulin administration. This suggests that metformin has the potential to ameliorate neurological deficits and enhance cognitive function in acute ischemic stroke patients with type 2 diabetes, likely by reducing oxidative stress levels, as evidenced by decreased levels of MAD and increased activity of SOD and GSH-Px [82]. Although numerous studies underscore the advantageous effects of metformin in the context of ischemic stroke, including the stimulation of neurogenesis [83,84] and angiogenesis [85,86,87], reduction of infarct volume [88,89], and enhancement of neurological recovery [90,91], some research indicates that the acute administration of metformin may exert potential adverse effects [92]; therefore, detailed investigations of its biodistribution, optimal timing for administration, and targeted mechanisms are imperative to advance its application as a neurotherapeutic agent [93].

Sinomenine (Sino) is an alkaloid derived from *Sinomenium acutum*, which exhibits anti-inflammatory effects and has been traditionally employed in China to treat neuralgia and rheumatic diseases [94]. Numerous studies have endeavored to elucidate the potential therapeutic application of Sino in ischemic stroke treatment. Qiu et al. [95] illustrated that Sino confers neuroprotection in ischemic stroke by suppressing NLRP3 inflammasomes through the AMPK pathway, both in vivo using a mouse model of middle cerebral artery occlusion (MCAO) and in vitro using an oxygen–glucose deprivation (OGD)-treated astrocytes/microglia model [95]. In a separate investigation, treatment with Sino significantly reduced cerebral infarction and neuronal apoptosis, lowered levels of inflammatory cytokines, and ameliorated neurological deficits in MCAO mice, possibly by inhibiting neuroinflammation through the CRYAB/STAT3 pathway [96]. Sino effectively mitigated cerebral damage and inflammation, while reinstating the equilibrium in cerebral oxidative stress, potentially via the Nuclear factor (erythroid-derived 2)-like 2 (Nrf2) signaling pathway [97].

Resveratrol (3,5,4′-trihydroxystilbene) is a natural compound classified as a stilbenoid, belonging to a group of polyphenolic compounds found in the skin of grapes, red wine, berries, and other nutrients [98]. Its neuroprotective effects have been validated in preclinical models of MCAO [99,100]. Pretreatment with resveratrol has improved oxidative stress markers and reduced the activities of antioxidant enzymes and Na^+^/K^+^–ATPase [101]. A different study proposed that the Smoothened (Smo) receptor could be a therapeutic target of resveratrol in order to help decrease microglial activity during the initial stage of a stroke [102]. Several studies investigated the role of resveratrol in the AMPK pathway. The results suggest that resveratrol offers neuroprotection via blocking phosphodiesterase (PDEs) and controlling the cAMP/AMPK/SIRT1 pathway, leading to decreased ATP energy usage in ischemic conditions [103]. Another study demonstrated that the protective effect of resveratrol partially relies on the activation of the AMPK/autophagy pathway, which was hindered by Compound C [104], possibly by activating JAK2/STAT3, thus upregulating the PI3K/AKT/mTOR pathway [105]. Despite the abundance of research in preclinical models demonstrating the neuroprotective effects of resveratrol, there are still constraints regarding the ideal dosage, treatment timing, and the selection of young and healthy animals [106]. Fodor et al. [107] studied the effect of resveratrol supplementation (100 and 200 mg/day, 12 months) in a group of patients who experienced stroke in the past 12 months and evidenced that they had overall better control of blood pressure, glycaemia, and lipid profile [107]. Also, patients who receive delayed r-tPA treatment show better treatment outcomes when resveratrol is given alongside compared to those who receive a placebo, as shown by improved NIHSS ratings [108].

N-methyl-D-aspartic acid (NMDA) is a water-soluble synthetic compound derived from amino acids that is commonly believed to enhance survival in the central nervous system [109]. Stimulation of synaptic NMDAR promotes the PI3K/Akt kinase pathway, leading to CREB (cAMP-response element binding protein)-dependent gene expression and repression of pro-death genes, ultimately promoting pro-survival effects [110]. In preclinical MCAO model studies, NMDAR antagonists shielded neurons from ischemic demise [111]; however, these findings have not successfully translated into clinical applications for acute stroke treatment [112]. In light of the limited clinical success observed with NMDA receptor antagonists, attention in stroke neuroprotection has shifted towards identifying descending intracellular signaling pathways activated by NMDARs [111]. In a study involving macaques, PSD-95 (postsynaptic density-95 protein) inhibitors, specifically Tat-NR2B9c, were utilized. This peptide consists of the nine carboxy-terminal amino acids of the N-methyl-D-aspartate receptor (NMDAR) NR2B subunit fused to the 11-mer HIV-1 Tat protein transduction domain. Its purpose was to uncouple postsynaptic density protein PSD-95 from neurotoxic signaling pathways, and it demonstrated that the treated group developed reduced infarct volumes [113]. A randomized, double-blind, controlled study across 14 hospitals in Canada and the USA used Tat-NR2B9c for individuals with either a ruptured or unruptured intracranial aneurysm suitable for endovascular repair. Although there was no discrepancy observed between the groups in terms of lesion volume detected by diffusion-weighted MRI, patients in the Tat-NR2B9c group experienced fewer ischemic infarcts compared to those in the placebo group [114].

**Figure 3:**
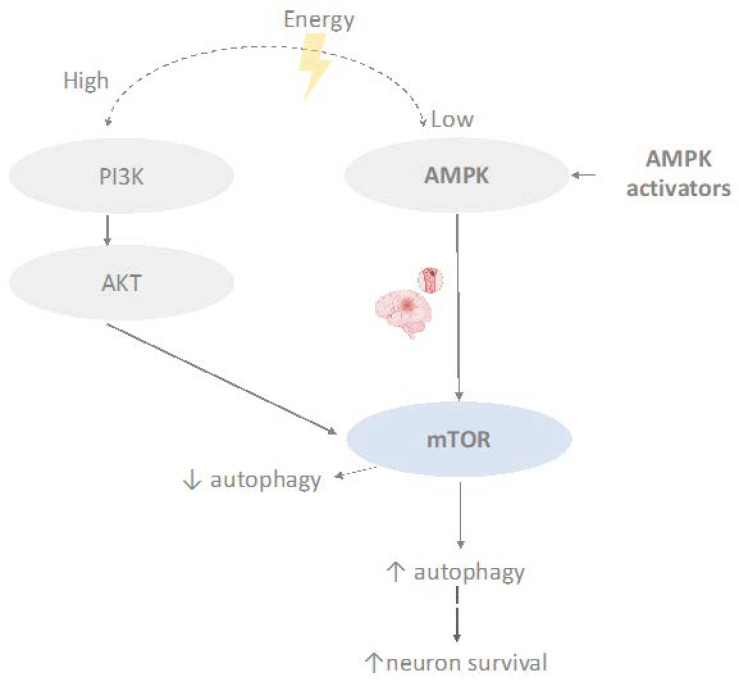
Overview of mTOR signaling pathway in ischemic stroke (created with BioRender.com (accessed on 10 March 2024)).

Therapeutic hypothermia (TH) involves the transient reduction of body temperature following acute cerebral ischemia to mitigate neuronal damage and enhance tissue resilience to restricted perfusion [115]. The inhibition of pAMPK due to hypothermia diminishes infarct volume, cerebral edema, and cerebral metabolic rate following middle cerebral artery occlusion (MCAO) in mice, with this protective effect further amplified by Compound C administration [116]. Several preclinical stroke models have demonstrated the protective effects of hypothermia, including reduced post-stroke inflammation [117], enhanced angiogenesis [118], decreased brain infarction and neurological deficits, lower levels of glycolytic enzymes [119], and reduced production of reactive oxygen species (ROS) [120]. However, conflicting findings suggest that this effect may be limited in aged animal models [121]. Another concern lies in establishing optimal perfusion conditions and ensuring procedural safety. In a rat model of middle cerebral artery occlusion (MCAO) and reperfusion, intra-arterial hypothermia induced with cold saline solution at 4 °C, via 2/3 RICA (0.50 mL/min) for 20 min, was found to be safe and feasible [122]. In clinical trials, conflicting results have been reported [123], likely due to small sample sizes, the time window, and varied protocols. For instance, the ICTuS-2 trial, despite being halted with only 120 enrolled patients, confirmed therapeutic hypothermia to be safe and feasible in acute ischemic stroke patients treated with rt-PA, albeit with a higher incidence of pneumonia mentioned [124]. The findings from another clinical trial indicate that there was no difference in the primary outcome between the groups, highlighting the need for improvement in the feasibility of the cooling procedure [125]. A subsequent follow-up of the study revealed elevated levels of Metalloproteinase-3 (MMP-3), fatty acid-binding protein (FABP), and interleukin-8 (IL-8) associated with hypothermia [126]. However, a more recent study found that combining mild hypothermia with remote ischemic preconditioning (RIPC) had a positive effect on brain protection, significantly reducing oxidative stress and associated inflammatory responses in 58 acute ischemic stroke patients [127]. The impact of the mTOR pathway on ischemic stroke and its potential therapeutic significance are depicted in Figure 3.

### 4.3. Secretome

Mesenchymal stromal cells (MSCs) are adult stem cells known for their self-renewal capacity and can be sourced from various tissues [128]. Despite their potential, MSC use poses challenges like ectopic tissue formation, host rejection, and pro-tumoral activities [129,130], yet they remain a focus in regenerative medicine due to their promise for tissue repair [131].

Recent attention has been drawn to MSCs as “trophic factories” because of their ability to secrete a plethora of bioactive molecules in response to their surroundings, collectively termed the MSC secretome [132]. Notably, MSC paracrine activities are gaining prominence, especially as their secretome has been observed to penetrate the blood–brain barrier, with only a fraction of intravenously administered MSCs reaching the brain [133,134,135,136], suggesting that their beneficial effects are predominantly mediated through paracrine signaling.

The MSC secretome comprises a wide range of molecules crucial for various biological processes [136], such as axonal growth [137], proliferation [138], apoptosis [139], and angiogenesis [140], as well as the release of genetic material like microRNAs (miRNAs) [141]. Stem cell secretome [142], along with small extracellular vesicles (sEVs) derived from MSCs have emerged as promising therapeutic agents [143].

Studies investigating MSC-derived extracellular vesicles (MSC-EVs) in ischemic stroke treatment have reported positive outcomes, including reductions in stroke volume [144,145], alleviation of neuroinflammation [145,146], and improvements in cognitive and motor function [147]. Enhanced neurogenesis and angiogenesis have also been observed in both adult and aged rat models [148]. Xin et al. [149] have demonstrated that exosomes originating from MSCs have the potential to transfer microRNA (miRNA)-133b to neuronal cells, leading to enhanced neurite outgrowth and improved functional recovery post-stroke [149]. Another potential mechanism underlying the neuroprotective effect of lymphocytes co-cultured with HCB-SCs (human cord blood-derived multipotent stem cells) in an MCAO rat model targets pyroptosis by promoting Tregs differentiation and suppressing NLRP3 inflammasome activation, ultimately reducing neuron apoptosis [150]. Moreover, numerous studies have elucidated the modulation of autophagy via the mTOR pathway. This includes both the inhibition of autophagy through pathways such as PI3K/Akt/mTOR [151,152] and PTEN/Akt/mTOR [153] as well as the inhibition of p53/Bnip3-mediated autophagy [154]. Conversely, autophagy can also be enhanced through mechanisms such as the attenuation of pyroptosis mediated by the NLRP3 inflammasome [155], or via pathways like BDNF/mTOR, PI3K/Akt/mTOR, and Notch2/mTOR, which are implicated in the regulation process of MSCs promoting autophagy [156].

**Figure 4:**
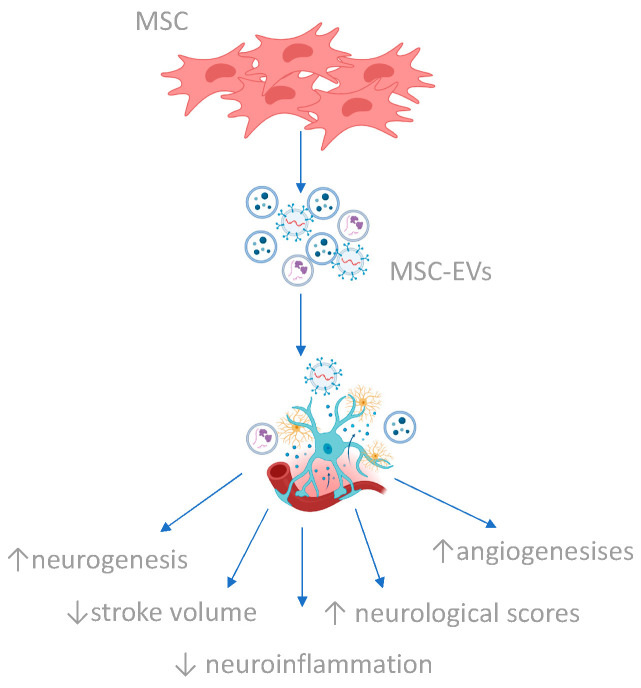
Overview of the secretome effects in ischemic stroke (created with BioRender.com (accessed on 10 March 2024)).

While there are currently no findings from clinical trials evaluating MSC secretome administration in stroke treatment, Dahbour et al. [157] explored the safety and efficacy of intrathecal administration of autologous bone marrow-derived MSCs (BM-MSCs) combined with their conditioned medium (CM) [157]. They observed a correlation between reduced brain lesions and increased levels of factors such as IL-6, IL-8, and VEGF in the CM. Despite minor adverse effects, the protocol was deemed safe, feasible, and potentially effective in stabilizing and reversing disease symptoms. Figure 4 illustrates the ramifications of the secretome in ischemic stroke, presenting its potential therapeutic applications. Table 1 provides a summarization of the therapies targeting the associated signaling pathways discussed.

## 5. Discussion

The RhoA/ROCK pathway emerges as a pivotal player in ischemic stroke, orchestrating diverse cellular responses that contribute to both injury and repair processes. Activation of RhoA triggers downstream signaling cascades mediated by ROCK, culminating in cytoskeletal rearrangements, cell contraction, and inflammation [158,159]. Despite its central role, targeting this pathway presents a double-edged sword, with both neuroprotective and detrimental effects observed.

On one hand, inhibition of ROCK has shown promise in preclinical models, demonstrating reductions in infarct size, improved neurological outcomes, and enhanced neurovascular remodeling [160,161]. These beneficial effects are attributed to ROCK inhibition-mediated vasodilation [162], suppression of inflammatory responses, and promotion of neuronal survival [162,163]. However, the translation of ROCK inhibitors into clinical practice faces hurdles, including off-target effects, variable efficacy, and challenges in achieving tissue-specific targeting [53].

The mTOR signaling pathway emerges as a central regulator of cellular homeostasis, exerting profound effects on cell growth, metabolism, and autophagy [164,165]. In the context of ischemic stroke, dysregulation of mTOR signaling contributes to neuronal injury, neuroinflammation, and impaired tissue repair.

Studies have demonstrated the dual role of mTOR in ischemic stroke pathophysiology, with both neuroprotective and neurotoxic effects observed depending on the context and timing of its activation. Activation of mTOR under physiological conditions promotes cell survival and neurogenesis [166], whereas dysregulated mTOR signaling in ischemic conditions exacerbates oxidative stress, inflammation, and neuronal death [167].

Targeting mTOR signaling for ischemic stroke treatment poses therapeutic challenges due to its intricate regulatory network and pleiotropic effects. While pharmacological modulation of mTOR holds promise for attenuating neuronal damage and promoting recovery [106], concerns regarding off-target effects, immunosuppression, and long-term safety necessitate cautious consideration [168].

Moreover, the interplay between mTOR and other signaling pathways, such as AMPK, autophagy, and neuroinflammation, adds complexity to therapeutic interventions. Strategies aimed at fine-tuning mTOR activity, restoring autophagic flux, and mitigating neuroinflammatory responses offer avenues for enhancing stroke outcomes while minimizing adverse effects.

The emergence of secretome-based therapies represents a paradigm shift in ischemic stroke treatment, harnessing the regenerative potential of mesenchymal stromal cells (MSCs) and their bioactive secreted factors. The MSC secretome encompasses a diverse array of cytokines, growth factors, and extracellular vesicles that exert neuroprotective, anti-inflammatory, and pro-regenerative effects [169].

Preclinical studies have demonstrated the efficacy of MSC secretome in attenuating neuronal damage, promoting tissue repair, and improving functional outcomes in ischemic stroke models [146,149]. Importantly, the paracrine actions of MSCs offer advantages over cell-based therapies, circumventing issues related to immune rejection, tumorigenicity, and logistical challenges associated with cell delivery [170].

Despite the promising preclinical data, the translation of secretome-based therapies into clinical practice faces several hurdles. Standardization of isolation and characterization protocols, optimization of dosing regimens, and validation of therapeutic efficacy in clinical trials are essential steps toward clinical implementation. Furthermore, efforts to establish regulatory frameworks, address manufacturing challenges, and ensure scalability are imperative for realizing the full therapeutic potential of MSC secretome in stroke management. Future research directions might involve elucidating the isoform-specific roles of ROCK and mTOR, exploring combinatorial approaches with other therapeutic modalities, and leveraging advanced drug delivery strategies to enhance specificity and efficacy.

Additionally, comorbidities represent a defining characteristic of stroke, exerting a dual impact by augmenting both the frequency of stroke occurrences and exacerbating resultant clinical outcomes [171]. These concurrent medical conditions, often present in individuals afflicted by stroke, contribute significantly to the complexity of the condition [172]. On the other hand, individuals maintaining a healthy lifestyle, characterized by abstaining from smoking, engaging in daily exercise, moderate alcohol consumption, and maintaining a moderate weight in their mid-forties, experienced a notably reduced risk of developing stroke [173] and neurodegenerative diseases compared to those with high-risk lifestyles [174,175]. In stroke research, prioritizing the inclusion of aged and comorbid animal models is paramount [176]. These models more faithfully replicate the clinical complexity observed in stroke patients, offering invaluable insights into the disease’s multifaceted nature, some studies underscore the varying efficacy of treatments in the aged brain, further emphasizing the importance of utilizing such models in stroke research [177,178,179,180]. These models more accurately reflect the clinical scenario seen in stroke patients, providing valuable insights into the efficacy and safety of potential therapeutic interventions.

Future directions should focus on refining experimental approaches, validating therapeutic targets, and translating preclinical findings into clinically effective interventions to address the significant burden of stroke worldwide.

## 6. Conclusions

In conclusion, our review underscores the critical roles of the RhoA/ROCK and mTOR signaling pathways in ischemic stroke pathophysiology, alongside the emerging potential of secretome-based therapies. These pathways and therapeutic approaches exhibit a delicate balance between beneficial and adverse effects, reflecting the complexity of ischemic stroke treatment. While the inhibition of ROCK and modulation of mTOR signaling present promising preclinical outcomes, their translation into clinical practice is hampered by challenges such as off-target effects and the need for precise targeting. Similarly, secretome-based therapies offer a novel, regenerative strategy for stroke treatment, yet face hurdles in standardization, dosing, and clinical validation.

## Figures and Tables

**Table 1:** Therapies targeting the associated signaling pathways implicated in stroke pathophysiology.

	Therapy	Targeting Pathway	Model	Species	Results	
RhoA/ROCK	Fasudil	ROCK inhibition	MCAO	Mice	↓ infarct size ↑ neurological deficit	[56]
	Fasudil	ROCK inhibition	Hemorrhage/reinfusion	Mice	↑ learning capacity	[57]
	Fasudil	ROCK inhibition	Ischemic stroke	Humans	↑ neurological status ↑ clinical outcome	[58]
mTOR						
	Metformin	AMPK activation	Global cerebral ischemia	Rats	↑ neuroprotection	[81]
	Metformin	↓ oxidative stress	Ischemic stroke and diabetes	Humans	↓ NIHSS	[82]
	Sinomenine	suppresses NLRP3 inflammasomes	MCAO	Mice	↑ neuroprotection	[95]
	Sinomenine	CRYAB/STAT3 pathway	MCAO	Mice	↓ cerebral infarction ↓ neuronal apoptosis ↑ neurological deficits	[96]
	Sinomenine	Nrf2 pathway	MCAO	Mice	↓ inflammation	[97]
	Resveratrol	cAMP/AMPK/SIRT1 pathway	MCAO	Rats	↑ neuroprotection	[103]
	Resveratrol	PI3K/AKT/mTOR pathway	MCAO	Rats	↓ neurological damage ↓ infarct volume	[105]
	Resveratrol		Ischemic stroke	Humans	Regulates blood pressure, glycemia and lipid profile	[107]
	Resveratrol	MMP-2, MMP-9	Ischemic stroke	Humans	↓ NIHSS	[108]
	Tat-NR2B9c	NI		Macaques	↓ infarct volume ↑ NHPSS	[113]
	Tat-NR2B9c	NI	Intracranial aneurysm	Humans	fewer ischemic infarcts	[114]
	Hypothermia	pAMPK inhibition	MCAO	Mice	↓ infarct volume	[116]
	Hypothermia	NI	Ischemic stroke	Humans	↑ incidence of pneumonia	[124]
	Hypothermia	NI	Ischemic stroke	Humans	No difference between groups	[125]
	Hypothermia	MMP-3, FABP, IL-8	Ischemic stroke	Humans	↑ MMP-3, FABP, IL-8	[126]
	Hypothermia + RIPC	NLRP3, MDA, SOD	Ischemic stroke	Humans	↓ oxidative stress ↓ inflammation ↓ NIHSS	[127]
Secretome						
	BMSC-Exos	NLRP3	MCAO	Rats	↓ infarct volume ↑ behavioral/cognitive deficit	[145]
	HCB-SCs	suppresses NLRP3 inflammasomes	MCAO	Rats	↓ neuronal apoptosis	[150]

RIPC—remote ischemic preconditioning; BMSC-Exos—bone marrow mesenchymal stem cell-derived exosomes; HCB-SCs—human cord blood-derived multipotent stem cells.

## Data Availability

The original contributions presented in the study are included in the review, further inquiries can be directed to the corresponding author.
